# Hyaluronan: A Neuroimmune Modulator in the Microbiota-Gut Axis

**DOI:** 10.3390/cells11010126

**Published:** 2021-12-31

**Authors:** Annalisa Bosi, Davide Banfi, Michela Bistoletti, Paola Moretto, Elisabetta Moro, Francesca Crema, Fabrizio Maggi, Evgenia Karousou, Manuela Viola, Alberto Passi, Davide Vigetti, Cristina Giaroni, Andreina Baj

**Affiliations:** 1 Department of Medicine and Surgery, University of Insubria, 21100 Varese, Italy; annalisa.bosi@uninsubria.it (A.B.); d.banfi3@studenti.uninsubria.it (D.B.); michela.bistoletti@gmail.com (M.B.); paola.moretto@unisubria.it (P.M.); fabrizio.maggi@uninsubria.it (F.M.); jenny.karousou@unisubria.it (E.K.); manuela.viola@uninsubria.it (M.V.); alberto.passi@uninsubria.it (A.P.); davide.vigetti@uninsubria.it (D.V.); andreina.baj@uninsubria.it (A.B.); 2 Department of Internal Medicine and Therapeutics, University of Pavia, 27100 Pavia, Italy; elisabetta.moro@unipv.it (E.M.); francesca.crema@unipv.it (F.C.); 3 Centre of Neuroscience, University of Insubria, 21100 Varese, Italy

**Keywords:** gastrointestinal tract, enteric nervous system, immune system, hyaluronan, gut microbiota

## Abstract

The commensal microbiota plays a fundamental role in maintaining host gut homeostasis by controlling several metabolic, neuronal and immune functions. Conversely, changes in the gut microenvironment may alter the saprophytic microbial community and function, hampering the positive relationship with the host. In this bidirectional interplay between the gut microbiota and the host, hyaluronan (HA), an unbranched glycosaminoglycan component of the extracellular matrix, has a multifaceted role. HA is fundamental for bacterial metabolism and influences bacterial adhesiveness to the mucosal layer and diffusion across the epithelial barrier. In the host, HA may be produced and distributed in different cellular components within the gut microenvironment, playing a role in the modulation of immune and neuronal responses. This review covers the more recent studies highlighting the relevance of HA as a putative modulator of the communication between luminal bacteria and the host gut neuro-immune axis both in health and disease conditions, such as inflammatory bowel disease and ischemia/reperfusion injury.

## 1. Introduction

The enteric microenvironment is a dynamic mosaic of different cellular “players” including enterocytes, neurons, enteric glial cells, immune cells, smooth muscle cells, interstitial cells of Cajal and immune cells, undergoing adaptation to maintain gut homeostasis [1]. Enterocytes, immune cells and neurons continuously receive and send stimuli to the commensal microbiota, and the existence of a microbiota−neuroimmune axis in the gut that retains a fundamental role in host health control is now proposed [2]. Alterations in the symbiotic interplay between the microbiota and the enteric microenvironment may have multiple consequences, including development of gut diseases, such as inflammatory bowel disease (IBD) and ischemia/reperfusion (I/R) injury, with high clinical and social impact. In this context, research aiming to uncover the mechanisms underlying modulation of the gut microbiota−neuroimmune axis in health conditions and, all the more, in the onset and/or development of gastrointestinal diseases, is compelling. In the gastrointestinal tract, the extracellular matrix (ECM) is constituted by a complex network of macromolecules that represent a structural support for resident cells, and may also interact with them and influence their functions [3]. More than 300 molecules concur to intestinal ECM formation, each of them with particular biochemical properties and physiological functions [4]. Among this vast array of ECM molecules, several data suggest that hyaluronan (HA), a glycosaminoglycan (GAG) that is a ubiquitous ECM component, may participate in the control of the epithelial, immune and neuronal functions by interacting with different cell types within the enteric microenvironment [5,6,7]. Furthermore, HA is also a modulator of gut microbiota homeostasis, retaining a metabolically relevant role for resident microorganisms and for modulating bacterial adhesiveness to the mucosal layer. This review encompasses a novel and comprehensive overview of the more recent evidence which suggests that HA represents a possible molecular player at the interface between commensal gut microbes and the gut neuroimmune system in health and disease conditions. This hypothesis is particularly interesting and fosters the possibility that modulation of HA homeostasis in the gut may help elucidate the pathogenesis of some important gut diseases, such as IBD and I/R injury, as well as to find innovative therapeutic approaches.

## 2. Hyaluronan: A Janus Face ECM Molecule for Tissue Homeostasis

HA is an unsulfated and unbranched glycosaminoglycan (GAG) devoid of a proteoglycan core protein. Thousands of disaccharide repeats (i.e., 2500 to 25,000 disaccharide units) of D-glucuronic acid (GlcUA) and N-acetyl-D-glucosamine (GlcNAc), bound through β1,3 and β1,4 glycosidic bonds, generate a linear polymer with high molecular weight (HMW), ranging between 1000 and 6000 kDa. HA has high hydrophilic properties, with every disaccharide monomer being able to interact with up to 15 water molecules, concurring to maintain tissue hydration, structure and elasticity [8]. HA is synthesized on cell surfaces as HMW polymers by HA synthases, namely HAS1, HAS2 and HAS3, which display different activity, regulation and tissue distribution, despite sharing similar amino acid sequences and structure. Among the three HASes, HAS2 retains the most important biological role and is critical for embryogenesis, since its deficiency results in embryonic lethality and failure of endocardial cushion formation, along with defects in yolk sac and vasculogenenesis [9,10]. The cytoplasmic availability and content of HA precursors, UDP-D-glucuronic acid (UDP-GlcA) and UDP-N-acetyl-D-glucosamine (UDP-GlcNAc) is critical for HA synthesis regulation. In addition to UDP-sugar availability, HA synthesis strongly depends on HAS2 expression, which is controlled at a post-transcriptional level and, as recently demonstrated, epigenetically, via the long noncoding RNA HAS2-AS1, a natural antisense transcript, inducing HAS2 expression after O-GlcNAcylation [11]. Endogenous HMW HA homeostasis and turnover are strictly dependent on mechanisms of HA degradation, occurring either enzymatically, via various classes of enzymes such as hyaluronidases (HYALs), procaryotic HA lyases, enzymes belonging to the Cell Migration-Inducing and hyaluronan-binding protein (CEMIP) family, and Transmembrane Protein 2 (TMEM2) [12], or as a consequence of oxidative/nitrosative processes. In pathological conditions, such as in cancer or during inflammation, degrading enzymes and Reactive oxygen species (ROS) may unbalance HMW HA tissue equilibrium towards the formation of low molecular weight HA (LMW) species having a molecular mass of <250 kDa, which can be further fragmented to yield short oligomers [13]. Six HYAL human isoforms, catalyzing the cleavage of the β-(1,4) linkage, have been recognized thus far, namely HYAL1-4, HYALP and PH-20 [14,15]. HYAL1 and HYAL2 represent the predominant isoforms and differ from each other for the dimensions of HA fragments generated and cellular localization: HYAL1 mediates the lysosomal HA degradation into small fragments of one to six disaccharides [15], while HYAL2 is located on cell membranes and produces larger fragments, which can be further degraded by HYAL1. During inflammation, sepsis, ischemia-reperfusion injury and cancer, the release of ROS or nitrogen species may produce small HA fragments, contributing to the development of tissue injury [16]. HA actions are mediated by interaction with several cellular targets, such as receptors like CD44, Receptor for Hyaluronan-Mediated Motility (RHAMM), Hyaluronic Acid Receptor for Endocytosis (HARE), Toll-Like Receptors (TLR2 and TLR4) and Tumor necrosis factor-Stimulated Gene 6 protein (TSG-6). CD44, the main HA receptor, is a ubiquitous transmembrane receptor that regulates a great variety of interactions between cells and the ECM, such as cell trafficking and aggregation [17]. In humans, the CD44 receptor is encoded by a single gene located on chromosome 11, containing 20 exons and generating 20 isoforms, of which a ubiquitous standard isoform is involved in the regulation of normal physiological functions, whereas other variants are predominantly expressed during cancer and inflammation [18]. The size of HA interacting with CD44 influences activation of downstream pathways, since HMW HA can stimulate CD44 clustering, while low molecular weight (LMW) HA fragments behave like antagonists of the receptor clustering strength, induced by the long polymer. HA molecular weight is also crucial for its interaction with TLR2 and TLR4. LMW HA fragments, by acting as Damage-Associated Molecular Patterns (DAMPs), are modulators of TLR2 and TLR4 receptors and are responsible for the formation of a unique receptor complex between CD44 and TLRs. During breast tumor invasion, the interaction between CD44 and TLR2/4 results in the release of proinflammatory chemokines via MyD88-nF-kB signaling [19]. Analogously, oligosaccharides made of 6-mers induce an inflammatory response by engaging both CD44 and TLR4 in human chondrocytes [20] and in neuron-like SH-SY5Y cells [21], while TLR2 and TLR4, but not CD44, are affected by 4-mer oligomers [22]. In dendritic cells, TLR4 can recognize fragments of 4-, 6- and 8-sugars, independently from TLR2, CD44 and RHAMM [23]. Macrophages isolated from either TLR4 or TLR2 knock-out mice are still capable of chemokine gene expression, after exposure to HA fragments, via a MyD88 downstream signaling pathway, whereas macrophages from TLR2/TLR4 double knockout mice completely lack the ability to express chemokines. Macrophage impaired ability to respond to HA fragments protects double TLR2/4 knockout mice from acute inflammation injury [24]. In contrast with the pro-inflammatory effects of LMW HA, HMW HA seems to have a positive anti-inflammatory effect in some clinical settings by modulating TLR-2 and TLR-4 cartilage expression, as shown in a model of experimental arthritis [25]. RHAMM is also a fundamental HA receptor, participating in tumor progression and metastasis (i.e., brain, colon, breast, prostate and endometrial cancer), osteoarthritis and bleomycin-induced lung injury. In spite of this pathogenetic involvement, RHAMM splice variants may be protective by conferring radiosensitivity to human breast cancer cell lines as well as by reducing HA-mediated inflammation [26,27,28,29,30,31,32]. RHAMM’s dual ability to promote tumorigenesis as well as epithelial−mesenchymal transition and wound healing depends on its interaction with other receptors, such as platelet-derived growth factor receptor (PDGFR), transforming growth factor beta receptor I (TGFβRI) and CD44 [17]. Although very few data are available at the moment on this issue, the actions of RHAMM may depend upon its interaction with HA of different molecular weight. For example, in fibrosarcoma cells, LMW HA may stimulate cell adhesion onto fibronectin, while HMW HA inhibits cell adhesion [33]. In addition, LMW HA may induce angiogenesis in epithelial cells during wound healing [34].

## 3. HA and Gut Microbiota

During the last decades, a considerable number of studies have shown that the microbial communities, collectively referred to as microbiota, harbored in the human body, may influence host health and disease states [7]. The gut microbiota constitutes the most abundant microbial community in the human body, consisting of about 3.8 × 10^13^ bacteria, 2–4 million genes, almost 2000 species and 7000 strains, besides virus, archaea, fungi and protozoa [35,36]. This complex microbial community has evolved along similar phylogenetic pathways within the host gut microenvironment, favoring the formation of a composite gut microbiota−mammalian host entity [37]. The gut microbiota plays a fundamental role in the maintenance of the host health by modulating immune responses, inducing defense mechanisms against pathogens and promoting the fermentation of indigestible dietary fibers, vitamin synthesis and drug metabolism [38,39]. Conversely, in the gastrointestinal tract, bacteria may benefit from a favorable nutritional and physical environment, suggesting that the coexistence between the commensal microbiota and host is the result of a dynamic equilibrium in which both get advantages. The gut is, indeed, one of the best examples of human body district where a complex interkingdom communication takes place via metabolites, cellular components, hormones, virulence factors and autoinducers that are released by prokaryotic and eukaryotic partners [7,40]. HA may take part in this crosstalk since it is essential for both gut homeostasis and gut-harbored microorganisms. Some bacteria, for example, can synthesize and use this GAG for structural, adhesion and nutritional purposes. One of the first pieces of evidence showing that some bacterial strains produce HA was shown by the study of Kendall, Heidelberger and Dawson, showing the isolation of a polysaccharide from the culture media of *Streptococcus haemolyticus* group A, which was successively identified as HA [41]. From a structural viewpoint, HA can be found in the capsule of other Streptococcus species belonging to group A and C [42,43,44] and in the capsule of Gram-negative *Pasteurella multocida* [45], allowing them to escape or overcome the host cellular and humoral immune responses since the capsule does not trigger the immune response of the host, prevents macrophage phagocytosis and enhances the virulence of these bacteria. HA concurring to the formation of *S. pyogenes* capsule is characterized by its HMW size and can be produced by incubation of cell-free membrane extracts of *S. pyogenes* with the UDP-sugars in the presence of divalent cations [46,47]. There are also studies suggesting that gut microbes can actively degrade HA produced by host enterocytes, as demonstrated for *Enterococcus faecium* isolated from human gut microbiota [48]. The mechanism has been described for *Streptococcus* spp., which are able to depolymerize HA to an unsaturated disaccharide via cell-surface hyaluronate lyase (HysA) [49]. Overall, these mechanisms are functional for pathogen bacteria to invade and colonize host tissue, causing tissue infection and damage [45]. Furthermore, HysA activity may contribute to produce potential carbon sources, such as glucose and glucuronic acid from the host’s ECM components available for bacterial metabolism [50]. HA degradation is also well conserved in the commensal genus Bacteroides, where *B. clarus* and *B. paurosaccharolyticus* actively catabolize chondroitin sulfate C and HA to yield nutritional energy. Human gut commensal bacteria, such as *Bacteroides* spp., *Bifidobacterium* spp., *Dialister* spp. and *Faecalibacterium* spp., metabolize HA in vitro, producing a significant amount of acetate, propionate and butyrate [51].

## 4. HA in the Host Gastrointestinal Tract

In the gastrointestinal tract, HA is involved in the regulation of the epithelial barrier as well as immune and neuronal functions. HA is abundantly produced by fibroblasts, smooth muscle cells, epithelial cells, neurons and immunocytes [4]. In the gut epithelium, HA exerts a key role in the maintenance of intestinal stem cell homeostasis through the interaction with other stabilizing ECM components. It is mainly expressed on the surface of intestinal stem cells and contains the binding sites for TLR modulation [52,53]. HA regulates epithelial stem cells also through its receptor CD44, which has long been considered a marker for stemness, and coordinates epithelial cell proliferation [54,55].

**Figure 1:**
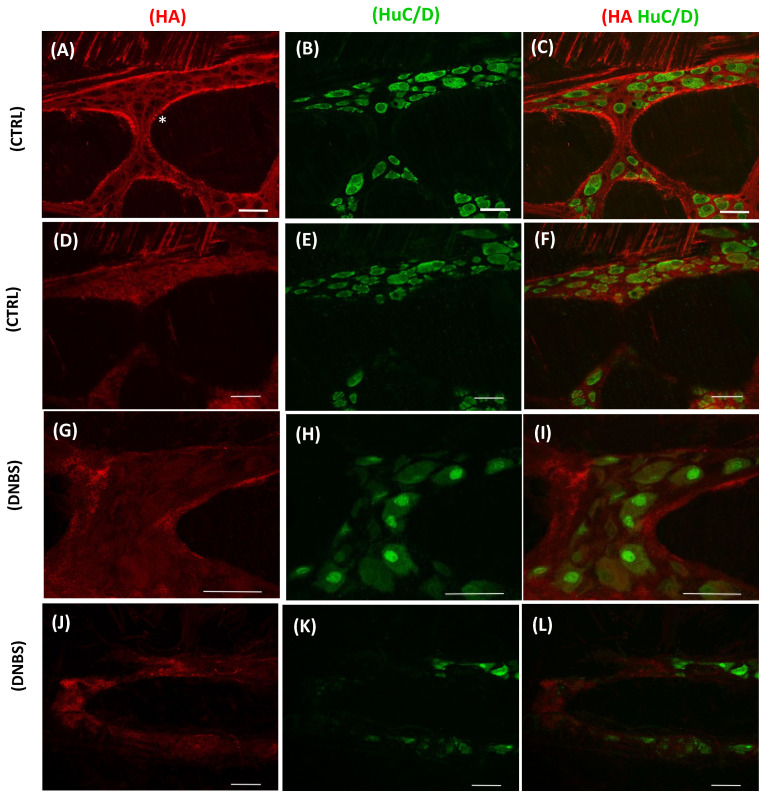
HA deposition in the rat colon myenteric plexus. (**A**–**F**) confocal image showing co-localization of HA with HuC/D (pan neuronal marker) in a median section of a colonic myenteric ganglion from control rats. HA was identified with a biotin-labeled HA-binding protein by streptavidin-FITC reaction. (**A**–**C**) Immunofluorescence was prevalently found in neuronal soma and in the perineuronal space. (**D**–**F**) HABP intensely stained the surface of the same ganglion and (**A**) interconnecting fibers (*). Bar 50 µm. (**G**–**L**) Confocal image showing co-localization of HA with HuC/D in a median section of a myenteric ganglion after DNBS-induced colitis in rats. (**G**–**I**) HABP fluorescence was prevalently found in the soma of myenteric neurons; perineuronal staining was absent. Several neurons displayed signs of distress with nuclear HuC/D translocation and faint cytoplasmic HuC/D immunoreactivity. Bar 100 µm. (**J**–**L**) HABP stained the surface of the same ganglion. Bar 50 µm (Adapted from Ref. [6]).

In both human and rodent models, HA deposition prevalently occurs beneath the gastrointestinal epithelium [6,56,57], where, owing to its high hydrophilic properties, it controls fluid exchange to and from blood vessels, and consequently, solute and nutrient absorption [58]. Besides modulating absorptive/secretory processes, as anticipated in the previous paragraph, HA has also important functions in the protection of the intestinal wall and in antibacterial defense. The gastrointestinal tract represents the major barrier surface of the body that is in contact with a non-sterile environment and is able to protect the host from pathogenic and harmful stimuli. From the outer to the inner layers, the gut microbiota, the mucosal lining and the epithelial and immune cells forming the gut-associated lymphoid tissue (GALT) accomplish this function. In this context, host cell HA is involved in multiple roles by modulating bacterial translocation, but also by promoting barrier integrity and immune system processes aiming to defend the host from the invasion of pathogens and/or commensal microorganisms. For example, this GAG is involved in epithelial cell production of natural antibiotic molecules, such as human β-defensin 2 (HBD2), released in the mucosal lining [59,60]. Defensins are a class of antimicrobial peptides acting against bacteria, fungus protozoa and viruses, distinct in α-defensins, which are produced by Paneth cells and neutrophils, and in β-defensins produced by epithelial cells [61]. Interestingly, HA purified from human milk enhanced the expression of human β-defensin 2 by epithelial cell lines in vitro and induced the expression of an orthologue of human β-defensin 2 in mice after oral administration [60]. High HA levels in human milk may favor the development of a healthy commensal microbial community during colonization of the sterile newborn gut [60,62]. Different studies reveal that dysregulation of defensin levels, occurring after direct exposure of the intestinal epithelium to microorganisms, correlates with development of inflammation [63,64]. HA-mediated production of human β-defensin 2 is common to different epithelial cells, including not only the intestinal epithelium but also the skin and vaginal epithelium, and involves TLR2 and TLR4 activation [59,65]. The effect of HA on human β-defensin 2 is dependent upon its molecular weight, since only a 35 kDa HA, and neither smaller not larger sized HA, may influence human β-defensin 2 production from epithelial cells in vitro [59]. In vivo oral administration of HA 35 kDa in mice has also been shown to protect from both *Citrobacter rodentium* and *Salmonella* infection by regulating the expression of tight junction proteins between intestinal epithelial cells, such as the cytoplasmic scaffolding proteins zona occludens (ZO-1) and claudin-2 [66,67]. Interestingly, no beneficial impact on tight junctions was observed in mice after intragastric administration of HMW HA of 2000 kDa, further strengthening the concept that the protective effect of HA on the epithelial barrier integrity is strictly dependent upon its molecular weight [59]. Due to the possible HA antimicrobial activity, several innovative GAG nanoparticle preparations have been produced in the field of the fight against pathogen microbes. In this context, elevated antimicrobial activity was demonstrated against *Staphylococcus aureus* and *Escherichia coli*, by HA complexed either with silver nanoparticles, or with heparin-reduced silver nanoparticles [68,69]. In view of its hydrophilic characteristics, HA has other important effects on the intestinal epithelium, contributing to local tissue hydration and lessening cell anchorage to the ECM, thus favoring cell migration and division. HA receptors can also link key proteins for cell activation, proliferation and migration via TSG-6 and the inter-α-inhibitor (IαI) [70]. In mice, in vivo intraperitoneal administration of HA 750 kDa promoted the proliferation of the colonic epithelium and the small intestine elongation via TLR4 and CD44 receptors [55]. Furthermore, the same treatment exerted a radioprotective effect on the intestinal epithelium, by increasing stem cell proliferation and reducing radiation-induced apoptosis [53]. Beneath the epithelium, a population of leukocytes comprising T- and B-lymphocytes, macrophages, plasma cells and mast cells reside in the *lamina propria*, where they provide immune protection from pathogenic organisms. In mice, in vivo intraperitoneal treatment with HA 750 kDa induced the activation of macrophages in the distal colon [71]. In accordance with this data, Asari and colleagues demonstrated that oral administration of 900 kDa HA controls immune responses during inflammation via TLR4. Since the epithelial barrier is impermeable to HMW HA [72], these latter effects may depend on the ability of this GAG to prevent bacterial translocation, more than on a direct action on the inflammatory process [73]. In the gut, HA is also involved in the modulation of the complex neuronal network composed of ganglia and interconnecting fiber strands, constituting the enteric nervous system (ENS). The ENS innervates smooth muscle and epithelial cells as well as intrinsic blood vessels and is involved in the control of different functions such as motility, gastric secretion, transport of fluids across the epithelium, blood flow and nutrient absorption, in a rather autonomous way with respect to the central nervous system (CNS) [74]. Several studies have provided evidence for the presence of an ECM molecule deposition composed of laminin, type IV collagen, nidogen, heparin sulfate proteoglycans and fibronectin, nearby the basement membrane that surrounds myenteric ganglia, but not within myenteric ganglia. However, recent data have shown the presence of HA within the myenteric plexus of the rat small intestine and colon [6,56] (Figure 1A–F).

In both rat colon preparations and in primary cultures of rat colon myenteric ganglia, myenteric neurons co-express HA and the neuronal marker HuC/D, suggesting that myenteric neurons represent a source for this GAG. Myenteric neurons produce HA via HAS2 and, to a minor extent, HAS1, contributing to the formation of a basal lamina surrounding myenteric ganglia, as well as a well-defined structure that occupies the perineuronal space between myenteric neurons. This latter structure resembles the perineuronal net (PNN) observed in the CNS, which controls neuronal communication, synaptic ion sorting and lateral protein mobility on the cell surface and protects neurons from oxidative stress and neurotoxins [75]. PNN disruption during neuroinflammation contributes to the pathogenesis of several neurological and neurodegenerative disorders, indicating that HA has a neuroprotective role favoring neuronal plasticity. This hypothesis has also been put forward for HA deposition within myenteric ganglia [76,77,78,79]. A distinctive feature of the enteric nervous system (ENS) is that enteric neurons communicate with different cell types within the enteric microenvironment, including immunocytes. Enteric neurons are located in close proximity to mucosal immunocytes and may regulate one another’s functions by releasing neurotransmitters, hormones and cytokines. Neuronal activation can lead to degranulation of mast cells and recruitment of neutrophils to the area. Neuropeptides released by enteric nerves, by interacting with their receptors located on lymphocytes, induce their differentiation and alter immunoglobulins production [80,81]. Interestingly, neutrophil recruitment on rat ileum myenteric ganglia significantly diminished after HA synthesis blockade with 4-metylumbelliferone (4-MU), suggesting that this GAG may play a role in the neuroimmune interplay between enteric neurons and immunocytes infiltrating the ENS [56]. The existence of an interplay between commensal bacteria and the ENS is also proposed [56]. For example, the ENS influences the microbiota composition both directly and indirectly, by controlling intestinal motility and allowing the removal of exuberant bacteria from the lumen [82,83]. Conversely, the enteric microbiota may influence the development of the ENS in critical postnatal life times, i.e., the first 3–4 years, when the density of enteric neurons may significantly decrease [84]. Furthermore, in mice, depletion of the commensal microbiota after chronic antibiotic treatment altered the structure and neurochemical coding of myenteric neurons and was associated with relevant changes in their neuromotor functions, which involved TLR2 activation on myenteric neurons and glial cells [85]. In view of the ability of HA to maintain both a healthy microbial gut flora and the integrity and function of myenteric neurons, a critical point, which however still needs to be explored, is the possible role of HA as a bridging molecule, directly connecting changes in commensal microorganisms with ENS alterations and vice versa.

## 5. HA and Inflammatory Bowel Disease

HA homeostasis and dynamics are influenced by the development of any pathological state involving ECM derangement. Among gastrointestinal disorders, numerous reports suggest that HA plays a role in the pathogenesis of inflammatory bowel diseases (IBD). IBD consists of two closely related disorders: Crohn’s disease (CD) and ulcerative colitis (UC), characterized by acute inflammatory flares of variable duration and frequency, followed by spontaneous or treatment-induced phases of remission [86]. CD manifests as a transmural inflammation injury developing along the whole gastrointestinal tract, while in UC the injury is limited to mucosa of the rectum and colon [87]. IBD pathogenesis still remains to be clarified, although both disorders seem to derive from an exaggerated immune response triggered by different stimuli deriving from pathogenetic microorganisms, the gut microbiota, or from the environment, in a genetically susceptible person [88,89]. In IBD patients, the main symptoms originate from alterations in secretory, sensory and motor intestinal functions, inducing diarrhea, abdominal pain, gastrointestinal bleeding and malnutrition [90]. The intestinal damage encompass a transitory acute phase associated with epithelial injury, altered secretion and enhanced visceral sensitivity, and by long-term alterations involving the neuromuscular motor function [6,91]. The crosstalk between different cellular populations of the gut microenvironment, including enterocytes, immunocytes and enteric neurons and the saprophytic microbiota underlays development of both the acute and chronic long-term changes [6,91,92].

The intestinal mucosa is a vast interface between the external environment and the host, whose integrity is fundamental for maintaining host’s health status. In disease states, i.e., during infection and inflammation, the loss of the epithelial barrier integrity and its consequent leakiness allows pathogenic bacteria translocation across the mucosal lining. As microorganisms and/or their by-products come in contact with cells of the enteric microenvironment, highly effective innate and adaptive immune mechanisms get into action, with consequent secretion of proinflammatory mediators, such as TNFα, IL-22 and IL-17, together with the activation of the inflammasome for host defense [7,93]. The hypothesis that particular microbial antigenic determinants may concur to the development of colitogenic changes in the intestinal mucosa associated with immune system dysfunction, leading to chronic inflammation, has been demonstrated in a vast number of preclinical studies on IBD [94,95,96,97].

**Figure 2:**
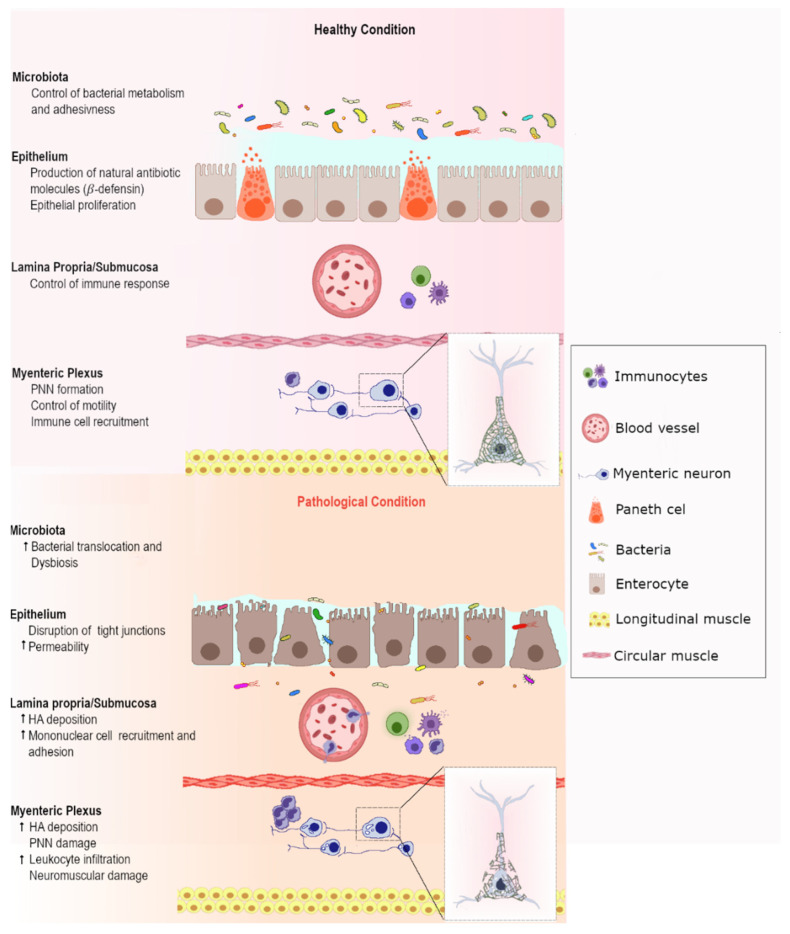
Schematic representation of HA-mediated effects on the gut microbiota and in the neuroimmune compartment in healthy and disease conditions. Abbreviations: HA: hyaluronan; PNN: perineuronal net.

In IBD, HA, by influencing different cellular players in the gut microenvironment, including epithelial, immune and neuronal cells, concurs to development of the inflammatory injury (Figure 2).

HA biosynthesis and deposition was observed in the submucosal and *muscularis propria* layers in both preclinical models of IBD and in IBD patients [57,98]. In IBD patients, HA levels are significantly higher in the inflamed colon compared to non-inflamed patient tissue or healthy controls [99]. From a structural viewpoint an inflammatory injury is associated with a rearrangement of HA distribution, consisting in a transition from a well-defined HA matrix to dense deposits in all intestinal layers, as observed in both dinitrobenzene sulfonic acid (DNBS)-treated rat colon and in dextran sulfate sodium (DSS)-treated mouse colon [6,57]. HA deposition probably occurs in the fibrotic process associated with inflammation, as observed in other peripheral organs and in vascular smooth muscle cells [52,100,101,102]. Indeed, the vascular endothelium is an important player in response to inflammatory stimuli, exhibiting an increased adhesion of immune cells in IBD, as suggested by the demonstration that microvascular endothelial cells isolated from the colon of IBD patients can produce a leukocyte adhesive HA matrix in vitro, which can be reproduced in endothelial cells obtained from non-IBD patients after stimulation with TNF-α and/or IL-1β [103,104,105]. HA synthesis is amplified by inflammatory or viral signals, such as TNF-α, yielding a highly adhesive HA matrix owing to the modification induced by TSG-6. TSG6 has a critical role in transferring the Heavy Chains (HCs) from IαI, a serum proteoglycan, onto HA to form a covalent, crosslinked HA-HC matrix [99,106,107]. From a functional viewpoint, the inflammation-induced HA-HC complex forms cable-like structures capable of spanning multiple cell lengths in order to recruit immunocytes [98,107]. Under normal in vitro conditions, resting leukocytes do not bind to HA in the pericellular matrix. However, after formation of HA-HC cable-like complexes in response to the synthetic analogue of double-stranded viral RNA, poly (I:C), leukocytes displayed strong binding capacity, suggesting that HA plays a role in their recruitment [98,108]. Monocyte adhesion to HA cable-like structures on colonic *muscularis mucosae* in response to inflammatory stimuli is also increased, further strengthening the concept that crosslinked HA potentially contributes to the inflammatory conditions. This phenomenon was observed both in vivo in a DSS murine model of colitis [57] and in vitro, using mucosal smooth muscle cells. Interestingly, IαI is associated and required for the formation of these structures [98]. In CD and UC patients, HA colonic accumulation in nonvascular space is in intimate contact with infiltrating leukocytes [98]. Recently, in a murine model of DSS-induced colitis, levels of serum-derived HA-associated protein (SHAP) have been positively related to TNFα serum levels as well as to the degree of colonic inflammation [109]. SHAP is an HA-binding protein, which covalently binds HA, via an ester linkage, and has been largely found in the hyperplastic *muscularis mucosae* of UC patients [110]. In an inflammatory condition, the presence of SHAP and leukocytes on endothelial cells suggested a possible key role for the protein SHAP in leukocyte recruitment and adhesion to the vascular endothelium, as already described in smooth muscle cells after the stimulation with poly (I:C) [98,111]. In mice, during DNBS-induced colitis, leukocyte recruitment following epithelial barrier disruption was subsequent to changes in HA deposition and distribution, suggesting that inflammation-induced changes in HA homeostasis precede immune cell recruitment during intestinal inflammation [57].

The increased deposition of HA in the gut after an intestinal injury depends upon enhanced HA synthesis, either in enzyme-increased activity or augmented expression. In particular, the expression of HAS2 and HAS3, but not of HAS1, increased in mouse colon epithelium after DSS-induced colitis [71]. Accordingly, in human intestinal microvessel endothelial cells and submucosal smooth muscle cells, HAS2 and HAS3 are upregulated in response to proinflammatory stimuli or stress stimuli [57,98]. In these studies, increased HAS expression was correlated with the property of HA to function as an adhesion molecule, released by microvessel endothelial cells and *muscularis mucosae* in order to recruit leukocytes via CD44 receptor activation [52]. Interestingly, experimental evidence shows that mice carrying a null deletion for HAS3 have highly decreased leukocyte infiltration in the inflamed colon that shows strikingly less inflammation and signs of tissue injury. HA degradation may be also influenced during inflammation. In a mouse model of colitis, examination of HYAL-1 and HYAL-2 tissue distribution indicates that these enzymes are expressed by different cellular populations: HYAL-1 is primarily expressed in smooth muscle and infiltrating leukocytes, while HYAL-2 expression is restricted to the endothelium and platelets [103]. In platelets, HYAL-2 is packaged within alpha-granules and, after activation, is translocated to the platelet surface. Platelet-mediated HA degradation is a fine-regulated process fundamental for the correct turnover of this GAG [112]. Of note is the ability of platelets in vitro to degrade HA matrix on the surface of TNF-α stimulated endothelial cells via HYAL-2 [103]. HA fragments derived from the platelet-mediated degradation can induce the release of IL-6 and IL-8 from naïve human peripheral blood monocytes. In IBD, platelet HYAL-2 levels are lower than in healthy individuals (on average 45% less), and this deficiency may contribute to HA accumulation within the microvasculature of the colon, thus exacerbating the inflammatory response [112]. Besides the canonical HYALs, other enzymes with the capability to degrade HA, such as the hyaluronan-degrading protein (CEMIP), also known as HYBID, TMEM2 and KIAA1199, are overexpressed in tissues isolated from CD. The overexpression of KIAA1199 in CD fibroblasts depends on IL-6 production, since its addition to control fibroblasts induced the expression of KIAA1199, while TNF-α had no effect on its gene expression. Fibroblasts isolated from CD patients secrete high IL-6 levels into their culture medium, boosting their own KIAA1199 expression [113]. There is evidence demonstrating that KIAA1199 is secreted from cultured fibroblasts and is deposited within the ECM. Furthermore, fibroblasts are able to degrade exogenous HA in vitro in a KIAA1199-dependent manner, since cells lacking this protein lose the ability to digest HA into smaller fragments [113,114]. The consequences of KIAA1199 overexpression during intestinal inflammation have not been clear-cut elucidated yet, although KIAA1199 activation conceivably generates endogenous danger signals in the form of HA fragments that perpetuate and promote inflammation and fibrosis in IBD. HA accumulation during an inflammatory injury involves also myenteric ganglia within the inner *muscularis propria* layer, as demonstrated in the rat colon myenteric plexus, after DNBS-induced colitis [6]. Such deposition depends upon an increased expression of HAS2, but not of HAS3 [6]. The increased inflammation-induced HA deposition on myenteric ganglia was associated with considerable changes in its distribution, with loss of the HA perineuronal structure, while the cytosolic and external basal lamina depositions were conserved (Figure 1G–L). During chronic inflammation, myenteric neurons show several signs of degeneration, including nuclear aggregates, vacuolization and smaller area, which are associated with changes in their excitability [91]. The loss of a protective perineuronal HA envelope probably contributes to the myenteric neuron derangement during an inflammatory challenge, as observed after degradation of the perineuronal net in cortical mouse slices [115]. As observed for HAS2 and HAS3 upregulation in the *lamina propria* microvessels, the increased HAS2 expression in myenteric neurons during intestinal inflammation may attract inflammatory cells towards myenteric ganglia, which display prominent leukocyte infiltration with respect to controls [6,92]. This hypothesis is strengthened by the ability of myenteric neuron cultures to produce HA “cable-like” structures similar to those observed in the *lamina propria* [6] (Figure 1C). In this context, HA may play a fundamental role in the interplay between myenteric neurons and immunocytes, which contributes to the remodeling of the neuronal network in response to a neuromuscular damage during gut inflammation [116]. Due to its central role in intestinal homeostasis, HA has been proposed as novel therapeutic strategy for the treatment of intestinal inflammation. Currently, many HA formulations are used worldwide for the treatment of pathological conditions such as bacterial acute rhinopharyngitis, eye surgery and osteoarthritis [117,118,119]. A few commercial intrarectal formulations that provide a protective barrier for intestinal epithelium are also available to promote mucosal healing and maintain gut microbial balance [120]. In a preclinical study, intracolonic administration of a combination of HA and mesalamine promoted wound healing in colonic injuries and inhibited myeloperoxidase (MPO) activity in the inflamed colon tissue of rats subjected to TNBS-induced colitis [121]. Moreover, intraperitoneal injection of HA successfully inhibited development of DSS-induced colitis in mice if administered at the initiation of DSS treatment. However, if administered in established colitic mice, HA was able to lower the severity of the disease in a TLR4- and COX-2-dependent manner [71]. Recently, new HA delivery strategies have been proposed to support conventional IBD therapy. A study demonstrated that HA-functionalized nanoparticle preparation protected against mucosal damage and reduced the expression of inflammatory cytokines in an in vitro model of gut inflammation. The same nanoparticle preparation, administered in a hydrogel formulation, was also able to prevent colonic neutrophilic infiltration and inflammatory cytokine expression after DSS-induced colitis [122]. Another promising HYAL-resistant nanoparticle, the bilirubin-conjugated HA nanoparticle (HABN), has been formulated by Lee and colleagues. HABN administration by oral gavage protected the colonic epithelium against pathological damage and suppressed immune cell activation in a murine model of DSS-induced colitis. Interestingly, HABN was also able to modulate the microbiota composition, improving bacterial richness of specific protective species, such as *Akkermansia muciniphila*, *Clostridium XIVα* and *Lactobacillus* [123].

## 6. HA and Intestinal Ischemia Reperfusion Injury

The intestine is one of the most sensitive organs to the damaging effects of ischemia/reperfusion (I/R) injury. I/R injury to the intestine occurs as a consequence of intestinal obstruction, shock, sepsis, vascular surgery, embolism, small bowel transplantation, necrotizing enterocolitis and IBD [124,125], thereby representing a major clinical problem associated with high mortality rates [125]. In this context, investigations on the pathophysiological mechanisms underlying intestinal I/R injury are compulsory since they could provide new insights into early onset detection and potential new therapeutic approaches. In the intestine, an acute ischemic insult is associated with epithelial barrier derangement, increased vascular permeability and bacterial translocation into the inner mucosa and submucosal layer [125,126]. Occlusion of the rat superior mesenteric artery followed by reperfusion was evaluated at different time points after ischemia. This induced changes in saprophytic bacterial communities, which consisted in an early growth of potentially pathogenetic strains, such as *Escherichia coli* and *Prevotella oralis*, and in proliferation of potentially protective bacteria, such as Lactobacilli, in later reperfusion phases, concomitantly with epithelial injury recovery [127]. Bacterial translocation towards distant organs, through the blood circulation, predisposes to development of systemic inflammation, respiratory failure, and multiple organ failure [125]. The crucial treatment to rescue intestinal tissue is to rapidly re-establish blood flow. However, the successive restoration of blood flow paradoxically initiates a cascade of events that may cause additional cell injury, exacerbating vascular and tissue damage [6,128], such as activation and adhesion of resident macrophages and neutrophil infiltration; release of ROS, proinflammatory chemokines such as TNF-α, interleukin 1 and 6 (IL-1 and IL-6) and activation of protein kinases [129,130]. After blood flow restoration, the lining epithelium undergoes *“restitutio ad integrum”*, while other cell types, such as myenteric neurons, may be compromised by the I/R injury, undergoing irreversible changes and, even, cell death [131]. The consequent neuronal loss may result in transit slowing and hampered food digestion, suggestive of enduring neuropathy [56,131]. Myenteric plexus adaptation may depend upon neuroimmune signaling since the abundance of eosinophils and mast cells in the *muscularis propria* and in the myenteric ganglia lasts for several weeks after the I/R damage [56,131]. In this context, HA may represent a possible molecular player favoring the interplay among different cell types within the enteric microenvironment [56]. In a recent study carried out in the rat, a temporary occlusion of the superior mesenteric artery causing I/R injury induced an accumulation of HA in the *muscularis propria*, on myenteric ganglia surface and in the perineuronal space [56]. HA deposition has been demonstrated to be strictly correlated with I/R injury in the CNS as well as in the peripheral tissues. Ahmed Al’Qteishat and colleagues demonstrated an enhanced deposition of total HA and LMW HA in post-mortem tissue and in the serum of patients subjected to ischemic stroke [132]. In this study, a significant upregulation of different molecular components related to HA homeostasis occurred. For example, increased expression and activity of HYALs favored LMW HA deposition in infiltrating mononuclear cells from stroke and peri-infarcted brain regions. In addition, HAS1, HAS2, CD44, TSG6 and RHAMM expressions were enhanced in infiltrating inflammatory cells, in microvessels and in neurons suggesting that, in spite of the LMW HA-mediated inflammatory response, activation of HA and/or oligo-HA-induced cellular signaling pathways in neurons and microvessels may favor remodeling processes by stimulating angiogenesis and revascularization, as well as the survival of susceptible neurons [132]. In a model of photothrombotic stroke lesion in the adult mouse cortex, enhancement of HA levels six weeks following the ischemic insult was associated with increased microglial, glial fibrillary acidic protein (GFAP)-positive cells in the peri-infarct area expressing RHAMM, suggesting that HA-mediated signaling may regulate these key cellular modulators of neuroinflammatory processes [133]. Increased deposition of total HA and LMW HA fragments was also observed in a peripheral model of lung ischemia in mice as a result of an increased HAS expression [134]. In this study, HA fragmentation was correlated with the occurrence of neovascularization. In ischemic kidneys from diabetic rats, renal HA content started to increase already 24 h after ischemia and remained significantly high for 1–8 weeks from I/R [135]. After an experimentally induced warm renal ischemia in mice, HA accumulation was observed in the cortex region, a structure where the presence of HA is not normally present [136]. Activation of HYALs seems to be mandatory to protect the kidneys from an ischemic injury, since in Hyal1^−/−^ and Hyal2^−/−^ knockout mice, unilateral I/R injury was more prominent and depended upon higher HA accumulation compared to wild-type mice [137].

As observed in the CNS and peripherally, in the rat small intestine, HA accumulation after I/R principally depended upon overexpression of HAS2, which was sensitive to the HA synthesis inhibitor 4-MU [56]. Of note is that 4-MU decreases HA production both by depleting UDP-glucuronic acid, required for HA synthesis, and by downregulating HAS2 [138]. In the rat small intestine, intraperitoneal 4-MU treatment was associated with reduced I/R-induced morphological and architectural alterations in some myenteric neurons and within the *muscularis propria,* as well as with a lower neutrophilic infiltration, suggesting a positive beneficial effect of the inhibitor on the intestinal neuromuscular compartment. In accordance with data obtained in the CNS after an ischemic stroke, such favorable effects may occur as a consequence of a reduced HA fragmentation underlaying development of neuroinflammation in the neuromuscular compartment [132]. As also suggested for the ischemic brain, a fine balance between HMW and LMW HA may be required to support the survival and function of specific neuronal populations in myenteric neuronal pathways, namely VIPergic and tachykinergic, since I/R-associated HA deposition in the myenteric-plexus and *muscularis propria* compartment seemed to favor remodeling processes sustaining intestinal functions [56,131]. Among the major receptor pathways involved in neuroimmune adaptation during intestinal disease states, TLR2 and TLR4 play an outstanding role, being able to modulate not only the responses to microbiota-derived metabolites in the innate immune system, but also in myenteric neurons and enteric glial cells [139,140]. In view of the ability of HA to influence TLR-mediated responses, HA can be considered as a bridging molecule in the gut-microbiota−neuroimmune axis, which participates in microbial, neutrophilic and neuromuscular responses during I/R injury (Figure 2) by modulating common TLR pathways.

## 7. HA and Probiotic Interaction: Implication for IBD Treatment

Probiotics are microorganisms similar to gut saprophytic bacteria and are used to correct microbiota alterations associated with a variety of disease states, including gastrointestinal, pancreatic, and liver disorders, but, to date, solid clinical data are restricted to the treatment of infections, functional gut disorders and IBD [141]. The most studied probiotics in clinical settings are represented by Lactobacillus, Bifidobacterium and Saccharomyces species, which are commercialized in a variety of over-the-counter or prescription formulations as packets, capsules or food supplements [141,142]. Several controlled trials have shown that probiotic microorganisms, including nonpathogenic *E. coli, S. boulardii*, Lactobacillus and Bifidobacterium, may be effective in maintaining remission in UC and in treating mild to moderate flare-ups [143]. It is likely that several mechanisms collectively operate to sustain the action of probiotics in IBD. Given the ascertained correlation existing between microbial dysbiosis and IBD pathogenesis [144], probiotics may prevent gut colonization by pathobionts, i.e., symbionts that under specific genetic or environmental conditions are able to promote pathology. Probiotics may hamper pathobiont attachment to the epithelium and may promote the production of molecules which inhibit pathogen replication, such as lactic, propionic and acetic acid, as well as bacteriocins. In addition, probiotics regulate host innate and adaptive immune system functions by influencing macrophage, natural killer and cytotoxic T-cell activation, as well as IgA production, cytokine-expression profile and by stimulating TLRs on both immune and enteric glial cells [145,146]. For example, VSL#3, a widely studied and commercialized combined preparation that contains eight strains of lactic-acid-producing bacteria (LAB), such as *L. plantarum*, *L. delbrueckii* subsp. bulgaricus, *L. casei*, *L. acidophilus*, *B. breve*, *B. longum*, *B. infantis* and *S. salivarius* subsp. thermophilus, has been shown to increase regulatory cytokines levels and to reduce pro-inflammatory cytokines, TLRs, NF-kB and TNFα expression in IBD [147]. Interestingly, in a mouse model of trinitrobenzene sulfonic (TNBS) acid-induced colitis, administration of *B. longum* HY8004 and *L. plantarum* AK8-4 exhibited the most potent effect in inhibiting inflammatory cytokine expression via TLR4 receptor-mediated NF-kB activation as well as in reducing colitis-associated intestinal bacterial degradation of HA and chondroitin sulfate [148]. Similar results were obtained after Lactobacillus plantarum HY115 and L. brevis HY7401 administration in mice subjected to dextran sulfate sodium (DSS)-induced colitis [149]. Accordingly, two plant-derived LAB strains displayed anti-inflammatory properties by producing exopolysaccharides, which prevented LMW HA production by HYAL [150]. Indeed, a reciprocal relationship seems to exist between probiotics and HA, which favors the stability of both bacteria and the GAG, since low HA concentrations in the presence of HYALs promoted LAB growth in vitro [151]. Conversely, some next-generation probiotics, such as Bacteroides, frequently detected in the human gut microbiota, showed a potent HA degrading ability which serves for both adhesive and nutrient purposes [152]. In view of the clinically favorable interplay between probiotics and HA, several probiotic formulations encapsulated with HA have been synthesized in order to ameliorate bacterial stability in the gastrointestinal tract. One of the major drawbacks of probiotic administration is certainly represented by the considerable loss of viability and bioactivity caused by elevated acidic and bile salt concentrations in the human gut. To overcome this challenge of the oral administration of live-probiotic, different encapsulation approaches have been proposed [48,153]. Encapsulation of *L. rhamnosus* in an HA-based hydrogel significantly enhanced the probiotic viability along the gastrointestinal tract and exerted better therapeutic effects against *Salmonella*-induced enteritis with respect to the free probiotic formulation [154]. LAB encapsulated with lyophilized HA showed more favorable effects in IBD patients by increasing mucous-strain adherence and by enhancing strain proliferation and viability [155]. Remarkably, a novel orally administered core-shell microsphere that targets the colon to collapse and release a thiolated-HA hydrogel core has demonstrated both in vivo and in vitro efficacy in reducing the inflammatory response, in alleviating DSS-induced colitis in mice and in augmenting probiotic bacterial communities, i.e., Lactobacillus and Bifidobacterium, with an overall benefit for the intestinal homeostasis and representing a promising candidate for IBD treatment [156].

## 8. Concluding Remarks

In the gastrointestinal tract, HA, one of the main well-studied ECM components, has multiple roles, comprising modulation of epithelial defense, immune responses and the neuromuscular function both in health and disease conditions. Such a modulatory role results from the activation of HA-dependent signaling pathways on epithelial, immune and neuronal cells within the enteric microenvironment as well as from the ability of the GAG to influence the stability of the symbiotic gut microbiota. Since a well-established interplay exists between microorganisms harboring the intestinal lumen, enteric neurons and immunocytes, HA may be in good reason considered a crucial molecular player at the interface between gut microbiota signals and neuroimmune responses. As also observed in other disease states, such as cancer and osteoarthritis, HA may participate in gut pathophysiological conditions, i.e., during IBD or I/R injury. The effects of HA in gastrointestinal disorders strongly depend on its fragmentation, being either beneficial or harmful according to the different molecular weights. A more clear-cut definition of the different mechanisms underpinning HA deposition and fragmentation in IBD and intestinal I/R injury are required to better elucidate its involvement in disease-related gut microbiota−neuroimmune axis alterations, thereby fostering new potential therapeutic approaches. This supports the possibility of administering exogenous HA, as already suggested in several clinical settings, including wound healing in patients with burns, trauma and ulcers or in osteoarthritis treatment. In this context, administration of exogenous HA may represent an adjuvant therapy for the treatment of digestive disorders characterized by dysbiosis and alteration of the neuroimmune axis, such as IBD and intestinal I/R injury.

## References

[1] Giaroni C., De Ponti F., Cosentino M., Lecchini S., Frigo G. (1999). Plasticity in the enteric nervous system. Gastroenterology.

[2] Pellegrini C., Antonioli L., Colucci R., Blandizzi C., Fornai M. (2018). Interplay among gut microbiota, intestinal mucosal barrier and enteric neuro-immune system: A common path to neurodegenerative diseases?. Acta Neuropathol..

[3] Manou D., Caon I., Bouris P., Triantaphyllidou I.E., Giaroni C., Passi A., Karamanos N.K., Vigetti D., Theocharis A.D. (2019). The complex interplay between extracellular matrix and cells in tissues. Methods in Molecular Biology.

[4] Pompili S., Latella G., Gaudio E., Sferra R., Vetuschi A. (2021). The Charming World of the Extracellular Matrix: A Dynamic and Protective Network of the Intestinal Wall. Front. Med..

[5] De La Motte C.A., Kessler S.P. (2015). The role of hyaluronan in innate defense responses of the intestine. Int. J. Cell Biol..

[6] Filpa V., Bistoletti M., Caon I., Moro E., Grimaldi A., Moretto P., Baj A., Giron M.C., Karousou E., Viola M. (2017). Changes in hyaluronan deposition in the rat myenteric plexus after experimentally-induced colitis. Sci. Rep..

[7] Bistoletti M., Bosi A., Banfi D., Cristina G., Baj A. (2020). The microbiota-gut-brain axis: Focus on the fundamental communication pathways. Progress in Molecular Biology and Translational Science.

[8] Hunger J., Bernecker A., Bakker H.J., Bonn M., Richter R.P. (2012). Hydration dynamics of hyaluronan and dextran. Biophys. J..

[9] Toole B.P. (2001). Hyaluronan in morphogenesis. Semin. Cell Dev. Biol..

[10] Camenisch T.D., Spicer A.P., Brehm-Gibson T., Biesterfeldt J., Augustine M.L., Calabro A., Kubalak S., Klewer S.E., McDonald J.A. (2000). Disruption of hyaluronan synthase-2 abrogates normal cardiac morphogenesis and hyaluronan-mediated transformation of epithelium to mesenchyme. J. Clin. Investig..

[11] Vigetti D., Deleonibus S., Moretto P., Bowen T., Fischer J.W., Grandoch M., Oberhuber A., Love D.C., Hanover J.A., Cinquetti R. (2014). Natural antisense transcript for hyaluronan synthase 2 (HAS2-AS1) induces transcription of HAS2 via protein O-GlcNAcylation. J. Biol. Chem..

[12] Tobisawa Y., Fujita N., Yamamoto H., Ohyama C., Irie F., Yamaguchi Y. (2021). The cell surface hyaluronidase TMEM2 is essential for systemic hyaluronan catabolism and turnover. J. Biol. Chem..

[13] Tavianatou A.G., Caon I., Franchi M., Piperigkou Z., Galesso D., Karamanos N.K. (2019). Hyaluronan: Molecular size-dependent signaling and biological functions in inflammation and cancer. FEBS J..

[14] Theocharis A.D., Skandalis S.S., Gialeli C., Karamanos N.K. (2016). Extracellular matrix structure. Adv. Drug Deliv. Rev..

[15] Bohaumilitzky L., Huber A.K., Stork E.M., Wengert S., Woelfl F., Boehm H. (2017). A trickster in disguise: Hyaluronan’s ambivalent roles in the matrix. Front. Oncol..

[16] Šoltés L., Mendichi R., Kogan G., Schiller J., Stankovská M., Arnhold J. (2006). Degradative action of reactive oxygen species on hyaluronan. Biomacromolecules.

[17] Misra S., Hascall V.C., Markwald R.R., Ghatak S. (2015). Interactions between hyaluronan and its receptors (CD44, RHAMM) regulate the activities of inflammation and cancer. Front. Immunol..

[18] Wu C.L., Chao Y.J., Yang T.M., Chen Y.L., Chang K.C., Hsu H.P., Shan Y.S., Lai M.D. (2015). Dual role of CD44 isoforms in ampullary adenocarcinoma: CD44s predicts poor prognosis in early cancer and CD44v is an indicator for recurrence in advanced cancer. BMC Cancer.

[19] Bourguignon L.Y.W., Wong G., Earle C.A., Xia W. (2011). Interraction of Low Molecular Weight Hyaluronan (LMW-HA) with CD44 and Tol-Like Receptor Promotes the Actin Filamnet-Associated Protein (AFAP-110)-Actin Binidng and MyD88-NFkB Signaling Leading to Pro-inflammatory Cytokine/Chenokine Production and Breast Tumor Invasion. Cytoskeleton.

[20] Campo G.M., Avenoso A., Campo S., D’Ascola A., Nastasi G., Calatroni A. (2010). Small hyaluronan oligosaccharides induce inflammation by engaging both toll-like-4 and CD44 receptors in human chondrocytes. Biochem. Pharmacol..

[21] Scuruchi M., D’Ascola A., Avenoso A., Campana S., Abusamra Y.A., Spina E., Calatroni A., Campo G.M., Campo S. (2016). 6-Mer Hyaluronan Oligosaccharides Modulate Neuroinflammation and α-Synuclein Expression in Neuron-Like SH-SY5Y Cells. J. Cell. Biochem..

[22] Termeer C., Benedix F., Sleeman J., Fieber C., Voith U., Ahrens T., Miyake K., Freudenberg M., Galanos C., Simon J.C. (2002). Oligosaccharides of hyaluronan activate dendritic cells via Toll-like receptor 4. J. Exp. Med..

[23] Termeer C.C., Hennies J., Voith U., Ahrens T., Weiss J.M., Prehm P., Simon J.C. (2000). Oligosaccharides of Hyaluronan Are Potent Activators of Dendritic Cells. J. Immunol..

[24] Jiang D., Liang J., Fan J., Yu S., Chen S., Luo Y., Prestwich G.D., Mascarenhas M.M., Garg H.G., Quinn D.A. (2005). Regulation of lung injury and repair by Toll-like receptors and hyaluronan. Nat. Med..

[25] Campo G.M., Avenoso A., Nastasi G., Micali A., Prestipino V., Vaccaro M., D’Ascola A., Calatroni A., Campo S. (2011). Hyaluronan reduces inflammation in experimental arthritis by modulating TLR-2 and TLR-4 cartilage expression. Biochim. Biophys. Acta-Mol. Basis Dis..

[26] Mele V., Sokol L., Kölzer V.H., Pfaff D., Muraro M.G., Keller I., Stefan Z., Centeno I., Terracciano L.M., Dawson H. (2017). The hyaluronan-mediated motility receptor RHAMM promotes growth, invasiveness and dissemination of colorectal cancer. Oncotarget.

[27] Schütze A., Vogeley C., Gorges T., Twarock S., Butschan J., Babayan A., Klein D., Knauer S.K., Metzen E., Müller V. (2016). RHAMM splice variants confer radiosensitivity in human breast cancer cell lines. Oncotarget.

[28] Korkes F., De Castro M.G., De Cassio Zequi S., Nardi L., Del Giglio A., De Lima Pompeo A.C. (2014). Hyaluronan-mediated motility receptor (RHAMM) immunohistochemical expression and androgen deprivation in normal peritumoral, hyperplasic and neoplastic prostate tissue. BJU Int..

[29] Rein D.T., Roehrig K., Schöndorf T., Lazar A., Fleisch M., Niederacher D., Bender H.G., Dall P. (2003). Expression of the hyaluronan receptor RHAMM in endometrial carcinomas suggests a role in tumour progression and metastasis. J. Cancer Res. Clin. Oncol..

[30] Tolg C., Hamilton S.R., Zalinska E., McCulloch L., Amin R., Akentieva N., Winnik F., Savani R., Bagli D.J., Luyt L.G. (2012). A RHAMM mimetic peptide blocks hyaluronan signaling and reduces inflammation and fibrogenesis in excisional skin wounds. Am. J. Pathol..

[31] Dunn S., Kolomytkin O.V., Waddell D.D., Marino A.A. (2009). Hyaluronan-binding receptors: Possible involvement in osteoarthritis. Mod. Rheumatol..

[32] Zaman A., Cui Z., Foley J.P., Zhao H., Grimm P.C., DeLisser H.M., Savani R.C. (2005). Expression and role of the hyaluronan receptor RHAMM in inflammation after bleomycin injury. Am. J. Respir. Cell Mol. Biol..

[33] Kouvidi K., Berdiaki A., Nikitovic D., Katonis P., Afratis N., Hascall V.C., Karamanos N.K., Tzanakakis G.N. (2011). Role of Receptor for Hyaluronic Acid-mediated Motility (RHAMM) in Low Molecular Weight Hyaluronan (LMWHA)-mediated fibrosarcoma cell adhesion. J. Biol. Chem..

[34] Gao F., Yang C.X., Mo W., Liu Y.W., He Y.Q. (2008). Hyaluronan oligosaccharides are potential stimulators to angiogenesis via RHAMM mediated signal pathway in wound healing. Clin. Investig. Med..

[35] Szigethy E., Levy-Warren A., Whitton S., Bousvaros A., Gauvreau K., Leichtner A.M., Beardslee W.R. (2004). Depressive Symptoms and Inflammatory Bowel Disease in Children and Adolescents: A Cross-Sectional Study. J. Pediatr. Gastroenterol. Nutr..

[36] Engström I. (1992). Mental Health and Psychological Functioning in Children and Adolescents with Inflammatory Bowel Disease: A Comparison with Children having Other Chronic Illnesses and with Healthy Children. J. Child Psychol. Psychiatry.

[37] Groussin M., Mazel F., Alm E.J. (2020). Co-evolution and Co-speciation of Host-Gut Bacteria Systems. Cell Host Microbe.

[38] Bergman E.N. (1990). Energy contributions of volatile fatty acids from the gastrointestinal tract in various species. Physiol. Rev..

[39] Kitamoto S., Nagao-Kitamoto H., Kuffa P., Kamada N. (2016). Regulation of virulence: The rise and fall of gastrointestinal pathogens. J. Gastroenterol..

[40] Bosi A., Banfi D., Bistoletti M., Giaroni C., Baj A. (2020). Tryptophan Metabolites Along the Microbiota-Gut-Brain Axis: An Interkingdom Communication System Influencing the Gut in Health and Disease. Int. J. Tryptophan Res..

[41] Kendall F.E., Heidelberger M., Dawson M.H. (1937). A Serologically Inactive Polysaccharide Elaborated by Mucoid Strains of Group a *Hemolytic streptococcus*. J. Biol. Chem..

[42] Seastone C.V. (1939). The virulence of Group C hemolytic Streptococci of animal origin. J. Exp. Med..

[43] Wessels M.R. (2019). Capsular Polysaccharide of Group A Streptococcus. Microbiol. Spectr..

[44] Kass E.H., Seastone C.V. (1944). The role of the mucoid polysaccharide (Hyaluronic acid) in the virulence of group A hemolytic Streptococci. J. Exp. Med..

[45] DeAngelis P.L., Jing W., Drake R.R., Achyuthan A.M. (1998). Identification and molecular cloning of a unique hyaluronan synthase from Pasteurella multocida. J. Biol. Chem..

[46] Cifonelli J.A., Dorfman A. (1957). The biosynthesis of hyaluronic acid by group A streptococcus. V. The uridine nucleotides of group A streptococcus. J. Biol. Chem..

[47] Sugahara K., Schwartz N.B., Dorfman A. (1979). Biosynthesis of hyaluronic acid by Streptococcus. J. Biol. Chem..

[48] Kawai K., Kamochi R., Oiki S., Murata K., Hashimoto W. (2018). Probiotics in human gut microbiota can degrade host glycosaminoglycans. Sci. Rep..

[49] Stern R., Jedrzejas M.J. (2006). Hyaluronidases: Their genomics, structures, and mechanisms of action. Chem. Rev..

[50] Berry A.M., Lock R.A., Thomas S.M., Rajan D.P., Hansman D., Paton J.C. (1994). Cloning and nucleotide sequence of the Streptococcus pneumoniae hyaluronidase gene and purification of the enzyme from recombinant *Escherichia coli*. Infect. Immun..

[51] Pan L., Ai X., Fu T., Ren L., Shang Q., Li G., Yu G. (2021). In vitro fermentation of hyaluronan by human gut microbiota: Changes in microbiota community and potential degradation mechanism. Carbohydr. Polym..

[52] De la Motte C.A. (2011). Hyaluronan in intestinal homeostasis and inflammation: Implications for fibrosis. Am. J. Physiol.-Gastrointest. Liver Physiol..

[53] Riehl T.E., Foster L., Stenson W.F. (2012). Hyaluronic acid is radioprotective in the intestine through a TLR4 and COX-2-mediated mechanism. Am. J. Physiol.-Gastrointest. Liver Physiol..

[54] Zohar R., Sodek J., McCulloch C.A.G. (1997). Characterization of stromal progenitor cells enriched by flow cytometry. Blood.

[55] Riehl T.E., Santhanam S., Foster L., Ciorba M., Stenson W.F. (2015). CD44 and TLR4 mediate hyaluronic acid regulation of Lgr5+ stem cell proliferation, crypt fission, and intestinal growth in postnatal and adult mice. Am. J. Physiol.-Gastrointest. Liver Physiol..

[56] Bistoletti M., Bosi A., Caon I., Chiaravalli A.M., Moretto P., Genoni A., Moro E., Karousou E., Viola M., Crema F. (2020). Involvement of hyaluronan in the adaptive changes of the rat small intestine neuromuscular function after ischemia/reperfusion injury. Sci. Rep..

[57] Kessler S., Rho H., West G., Fiocchi C., Drazba J., de la Motte C. (2008). Hyaluronan (HA) deposition precedes and promotes leukocyte recruitment in intestinal inflammation. Clin. Transl. Sci..

[58] Kvietys P.R., Granger D.N. (2010). Role of intestinal lymphatics in interstitial volume regulation and transmucosal water transport. Ann. N. Y. Acad. Sci..

[59] Hill D.R., Kessler S.P., Rho H.K., Cowman M.K., De La Motte C.A. (2012). Specific-sized hyaluronan fragments promote expression of human β-defensin 2 in intestinal epithelium. J. Biol. Chem..

[60] Hill D.R., Rho H.K., Kessler S.P., Amin R., Homer C.R., McDonald C., Cowman M.K., De La Motte C.A. (2013). Human milk hyaluronan enhances innate defense of the intestinal epithelium. J. Biol. Chem..

[61] Gallo R.L., Hooper L.V. (2012). Epithelial antimicrobial defence of the skin and intestine. Nat. Rev. Immunol..

[62] Coppa G.V., Gabrielli O., Buzzega D., Zampini L., Galeazzi T., MacCari F., Bertino E., Volpi N. (2011). Composition and structure elucidation of human milk glycosaminoglycans. Glycobiology.

[63] Swidsinski A., Loening-Baucke V., Theissig F., Engelhardt H., Bengmark S., Koch S., Lochs H., Dörffel Y. (2007). Comparative study of the intestinal mucus barrier in normal and inflamed colon. Gut.

[64] Wehkamp J., Koslowski M., Wang G., Stange E.F. (2008). Barrier dysfunction due to distinct defensin deficiencies in small intestinal and colonic crohn’ s disease. Mucosal Immunol..

[65] Gariboldi S., Palazzo M., Zanobbio L., Selleri S., Sommariva M., Sfondrini L., Cavicchini S., Balsari A., Rumio C. (2008). Low Molecular Weight Hyaluronic Acid Increases the Self-Defense of Skin Epithelium by Induction of β-Defensin 2 via TLR2 and TLR4. J. Immunol..

[66] Kim Y., Kessler S.P., Obery D.R., Homer C.R., McDonald C., de la Motte C.A. (2017). Hyaluronan 35 kDa treatment protects mice from Citrobacter rodentium infection and induces epithelial tight junction protein ZO-1 in vivo. Matrix Biol..

[67] Kessler S.P., Obery D.R., Nickerson K.P., Petrey A.C., McDonald C., de la Motte C.A. (2018). Multifunctional Role of 35 Kilodalton Hyaluronan in Promoting Defense of the Intestinal Epithelium. J. Histochem. Cytochem..

[68] Abdel-Mohsen A.M., Hrdina R., Burgert L., Abdel-Rahman R.M., Hašová M., Šmejkalová D., Kolář M., Pekar M., Aly A.S. (2013). Antibacterial activity and cell viability of hyaluronan fiber with silver nanoparticles. Carbohydr. Polym..

[69] Kemp M.M., Kumar A., Clement D., Ajayan P., Mousa S., Linhardt R.J. (2009). Hyaluronan- and heparin-reduced silver nanoparticles with antimicrobial properties. Nanomedicine.

[70] Chen W.Y.J., Abatangelo G. (1999). Functions of hyaluronan in wound repair. Wound Repair Regen..

[71] Zheng L., Riehl T.E., Stenson W.F. (2009). Regulation of Colonic Epithelial Repair in Mice by Toll-Like Receptors and Hyaluronic Acid. Gastroenterology.

[72] Balogh L., Polyak A., Mathe D., Kiraly R., Thuroczy J., Terez M., Janoki G., Ting Y., Bucci L.R., Schauss A.G. (2008). Absorption, uptake and tissue affinity of high-molecular-weight hyaluronan after oral administration in rats and dogs. J. Agric. Food Chem..

[73] Asari A., Kanemitsu T., Kurihara H. (2010). Oral administration of high molecular weight hyaluronan (900 kDa) controls immune system via toll-like receptor 4 in the intestinal epithelium. J. Biol. Chem..

[74] Baj A., Bistoletti M., Bosi A., Moro E., Giaroni C., Crema F. (2019). Marine Toxins and Nociception: Potential Therapeutic Use in the Treatment of Visceral Pain Associated with Gastrointestinal Disorders. Toxins.

[75] Van’t Spijker H.M., Kwok J.C.F. (2017). A Sweet Talk: The Molecular Systems of Perineuronal Nets in Controlling Neuronal Communication. Front. Integr. Neurosci..

[76] Carstens K.E., Lustberg D.J., Shaughnessy E.K., McCann K.E., Alexander G.M., Dudek S.M. (2021). Perineuronal net degradation rescues CA2 plasticity in a mouse model of Rett syndrome. J. Clin. Investig..

[77] Miyata S., Nishimura Y., Nakashima T. (2007). Perineuronal nets protect against amyloid beta-protein neurotoxicity in cultured cortical neurons. Brain Res..

[78] Pantazopoulos H., Woo T.U.W., Lim M.P., Lange N., Berretta S. (2010). Extracellular matrix-glial abnormalities in the amygdala and entorhinal cortex of subjects diagnosed with schizophrenia. Arch. Gen. Psychiatry.

[79] McRae P.A., Porter B.E. (2012). The perineuronal net component of the extracellular matrix in plasticity and epilepsy. Neurochem. Int..

[80] Giaroni C. (2015). Purinergic signalling and development of the autonomic nervous system. Auton. Neurosci..

[81] Lakhan S.E., Kirchgessner A. (2010). Gut inflammation in chronic fatigue syndrome. Nutr. Metab..

[82] Rolig A.S., Mittge E.K., Ganz J., Troll J.V., Melancon E., Wiles T.J., Alligood K., Stephens W.Z., Eisen J.S., Guillemin K. (2017). The enteric nervous system promotes intestinal health by constraining microbiota composition. PLoS Biol..

[83] Hyland N.P., Cryan J.F. (2016). Microbe-host interactions: Influence of the gut microbiota on the enteric nervous system. Dev. Biol..

[84] Collins J., Borojevic R., Verdu E.F., Huizinga J.D., Ratcliffe E.M. (2014). Intestinal microbiota influence the early postnatal development of the enteric nervous system. Neurogastroenterol. Motil. Off. J. Eur. Gastrointest. Motil. Soc..

[85] Caputi V., Marsilio I., Filpa V., Cerantola S., Orso G., Bistoletti M., Paccagnella N., De Martin S., Montopoli M., Dall’Acqua S. (2017). Antibiotic-induced dysbiosis of the microbiota impairs gut neuromuscular function in juvenile mice. Br. J. Pharmacol..

[86] Ng S.C., Shi H.Y., Hamidi N., Underwood F.E., Tang W., Benchimol E.I., Panaccione R., Ghosh S., Wu J.C.Y., Chan F.K.L. (2017). Worldwide incidence and prevalence of inflammatory bowel disease in the 21st century: A systematic review of population-based studies. Lancet.

[87] Lees C.W., Barrett J.C., Parkes M., Satsangi J. (2011). New IBD genetics: Common pathways with other diseases. Gut.

[88] Ni J., Wu G.D., Albenberg L., Tomov V.T. (2017). Gut microbiota and IBD: Causation or correlation?. Nat. Rev. Gastroenterol. Hepatol..

[89] Sekirov I., Russell S.L., Caetano M., Antunes L., Finlay B.B. (2010). Gut microbiota in health and disease. Physiol. Rev..

[90] Lomax A.E., Fernandez E., Sharkey K.A. (2005). Plasticity of the enteric nervous system during intestinal inflammation. Neurogastroenterol. Motil..

[91] Brierley S.M., Linden D.R. (2014). Neuroplasticity and dysfunction after gastrointestinal inflammation. Nat. Reviews. Gastroenterol. Hepatol..

[92] Bistoletti M., Micheloni G., Baranzini N., Bosi A., Conti A., Filpa V., Pirrone C., Millefanti G., Moro E., Grimaldi A. (2020). Homeoprotein OTX1 and OTX2 involvement in rat myenteric neuron adaptation after DNBS-induced colitis. PeerJ.

[93] Cheng H.Y., Ning M.X., Chen D.K., Ma W.T. (2019). Interactions between the gut microbiota and the host innate immune response against pathogens. Front. Immunol..

[94] Roy U., Gálvez E.J.C., Iljazovic A., Lesker T.R., Błażejewski A.J., Pils M.C., Heise U., Huber S., Flavell R.A., Strowig T. (2017). Distinct Microbial Communities Trigger Colitis Development upon Intestinal Barrier Damage via Innate or Adaptive Immune Cells. Cell Rep..

[95] Kullberg M.C., Andersen J.F., Gorelick P.L., Caspar P., Suerbaum S., Fox J.G., Cheever A.W., Jankovic D., Sher A. (2003). Induction of colitis by a CD4+ T cell clone specific for a bacterial epitope. Proc. Natl. Acad. Sci. USA.

[96] Garrett W.S., Lord G.M., Punit S., Lugo-Villarino G., Mazmanian S.K.K., Ito S., Glickman J.N., Glimcher L.H. (2007). Communicable Ulcerative Colitis Induced by T-bet Deficiency in the Innate Immune System. Cell.

[97] Hu B., Elinav E., Huber S., Strowig T., Hao L., Hafemann A., Jin C., Eisenbarth S.C., Flavell R.A. (2013). Microbiota-induced activation of epithelial IL-6 signaling links inflammasome-driven inflammation. Proc. Natl. Acad. Sci. USA.

[98] De la Motte C.A., Hascall V.C., Drazba J., Bandyopadhyay S.K., Strong S.A. (2003). Mononuclear leukocytes bind to specific hyaluronan structures on colon mucosal smooth muscle cells treated with polyinosinic acid: Polycytidylic acid. Inter-α-trypsin inhibitor is crucial to structure and function. Am. J. Pathol..

[99] De La Motte C.A., Hascall V.C., Calabro A., Yen-Lieberman B., Strong S.A. (1999). Mononuclear leukocytes preferentially bind via CD44 to hyaluronan on human intestinal mucosal smooth muscle cells after virus infection or treatment with poly(I·C). J. Biol. Chem..

[100] Viola M., Bartolini B., Vigetti D., Karousou E., Moretto P., Deleonibus S., Sawamura T., Wight T.N., Hascall V.C., De Luca G. (2013). Oxidized low density lipoprotein (LDL) affects hyaluronan synthesis in human aortic smooth muscle cells. J. Biol. Chem..

[101] Vigetti D., Rizzi M., Moretto P., Deleonibus S., Dreyfuss J.M., Karousou E., Viola M., Clerici M., Hascall V.C., Ramoni M.F. (2011). Glycosaminoglycans and glucose prevent apoptosis in 4-methylumbelliferone- treated human aortic smooth muscle cells. J. Biol. Chem..

[102] Moretto P., Karousou E., Viola M., Caon I., D’Angelo M.L., De Luca G., Passi A., Vigetti D. (2015). Regulation of hyaluronan synthesis in vascular diseases and diabetes. J. Diabetes Res..

[103] De La Motte C., Nigro J., Vasanji A., Rho H., Kessler S., Bandyopadhyay S., Danese S., Fiocchi C., Stern R. (2009). Platelet-derived hyaluronidase 2 cleaves hyaluronan into fragments that trigger monocyte-mediated production of proinflammatory cytokines. Am. J. Pathol..

[104] Deban L., Correale C., Vetrano S., Malesci A., Danese S. (2008). Multiple pathogenic roles of microvasculature in inflammatory bowel disease: A jack of all trades. Am. J. Pathol..

[105] Caravà E., Moretto P., Caon I., Parnigoni A., Passi A., Karousou E., Vigetti D., Canino J., Canobbio I., Viola M. (2021). Ha and hs changes in endothelial inflammatory activation. Biomolecules.

[106] Rugg M.S., Willis A.C., Mukhopadhyay D., Hascall V.C., Fries E., Fülöp C., Milner C.M., Day A.J. (2005). Characterization of complexes formed between TSG-6 and inter-α- inhibitor that act as intermediates in the covalent transfer of heavy chains onto hyaluronan. J. Biol. Chem..

[107] Milner C.M., Tongsoongnoen W., Rugg M.S., Day A.J. (2007). The molecular basis of inter-α-inhibitor heavy chain transfer on to hyaluronan. Biochem. Soc. Trans..

[108] Culty M., O’Mara T.E., Underhill C.B., Yeager H., Swartz R.P. (1994). Hyaluronan receptor (CD44) expression and function in human peripheral blood monocytes and alveolar macrophages. J. Leukoc. Biol..

[109] Yamaguchi Y., Noda H., Okaniwa N., Adachi K., Shinmura T., Nakagawa S., Ebi M., Ogasawara N., Funaki Y., Zhuo L. (2017). Serum-Derived Hyaluronan-Associated Protein Is a Novel Biomarker for Inflammatory Bowel Diseases. Digestion.

[110] Zhao M., Yoneda M., Ohashi Y., Kurono S., Iwata H., Ohnuki Y., Kimata K. (1995). Evidence for the covalent binding of SHAP, heavy chains of inter-alpha-trypsin inhibitor, to hyaluronan. J. Biol. Chem..

[111] Wilkinson T.S., Potter-Perigo S., Tsoi C., Altman L.C., Wight T.N. (2004). Pro- and anti-inflammatory factors cooperate to control hyaluronan synthesis in lung fibroblasts. Am. J. Respir. Cell Mol. Biol..

[112] Albeiroti S., Ayasoufi K., Hill D.R., Shen B., de la Motte C.A. (2015). Platelet hyaluronidase-2: An enzyme that translocates to the surface upon activation to function in extracellular matrix degradation. Blood.

[113] Sarmento O.F., Svingen P.A., Xiong Y., Xavier R.J., McGovern D., Smyrk T.C., Papadakis K.A., Urrutia R.A., Faubion W.A. (2015). A novel role for KLF14 in T regulatory cell differentiation. Cell. Mol. Gastroenterol. Hepatol..

[114] Yoshida H., Nagaoka A., Kusaka-Kikushima A., Tobiishi M., Kawabata K., Sayo T., Sakai S., Sugiyama Y., Enomoto H., Okada Y. (2013). KIAA1199, a deafness gene of unknown function, is a new hyaluronan binding protein involved in hyaluronan depolymerization. Proc. Natl. Acad. Sci. USA.

[115] Shah A., Lodge D.J. (2013). A loss of hippocampal perineuronal nets produces deficits in dopamine system function: Relevance to the positive symptoms of schizophrenia. Transl. Psychiatry.

[116] Lakhan S.E., Kirchgessner A. (2010). Neuroinflammation in inflammatory bowel disease. J. Neuroinflamm..

[117] Varricchio A., Capasso M., Avvisati F., Varricchio A.M., De Lucia A., Brunese F.P., Ciprandi G. (2014). Inhaled hyaluronic acid as ancillary treatment in children with bacterial acute rhinopharyngitis. J. Biol. Regul. Homeost. Agents.

[118] Tashiro T., Seino S., Sato T., Matsuoka R., Masuda Y., Fukui N. (2012). Oral administration of polymer hyaluronic acid alleviates symptoms of knee osteoarthritis: A double-blind, placebo-controlled study over a 12-month period. Sci. World J..

[119] Balazs E.A. (2008). Hyaluronan as an ophthalmic viscoelastic device. Curr. Pharm. Biotechnol..

[120] Kotla N.G., Bonam S.R., Rasala S., Wankar J., Bohara R.A., Bayry J., Rochev Y., Pandit A. (2021). Recent advances and prospects of hyaluronan as a multifunctional therapeutic system. J. Control. Release Off. J. Control. Release Soc..

[121] Chiu C.-T., Kuo S.-N., Hung S.-W., Yang C.-Y. (2017). Combined Treatment with Hyaluronic Acid and Mesalamine Protects Rats from Inflammatory Bowel Disease Induced by Intracolonic Administration of Trinitrobenzenesulfonic Acid. Molecules.

[122] Xiao B., Zhang Z., Viennois E., Kang Y., Zhang M., Han M.K., Chen J., Merlin D. (2016). Combination Therapy for Ulcerative Colitis: Orally Targeted Nanoparticles Prevent Mucosal Damage and Relieve Inflammation. Theranostics.

[123] Lee Y., Sugihara K., Gillilland M.G., Jon S., Kamada N., Moon J.J. (2020). Hyaluronic acid–bilirubin nanomedicine for targeted modulation of dysregulated intestinal barrier, microbiome and immune responses in colitis. Nat. Mater..

[124] Practice C., Committee P.E. (2003). American Gastroenterological Association medical position statement: Guidelines on osteoporosis in gastrointestinal diseases. Gastroenterology.

[125] Haglund U., Bergqvist D. (1999). Intestinal ischemia—The basics. Langenbecks Arch. Surg..

[126] Nadatani Y., Watanabe T., Shimada S., Otani K., Tanigawa T., Fujiwara Y. (2018). Microbiome and intestinal ischemia/reperfusion injury. J. Clin. Biochem. Nutr..

[127] Wang F., Li Q., Wang C., Tang C., Li J. (2012). Dynamic alteration of the colonic microbiota in intestinal ischemia-reperfusion injury. PLoS ONE.

[128] Mallick I.H., Yang W., Winslet M.C., Seifalian A.M. (2004). Ischemia-reperfusion injury of the intestine and protective strategies against injury. Dig. Dis. Sci..

[129] Giaroni C., Zanetti E., Giuliani D., Oldrini R., Marchet S., Moro E., Borroni P., Trinchera M., Crema F., Lecchini S. (2011). Protein kinase c modulates NMDA receptors in the myenteric plexus of the guinea pig ileum during in vitro ischemia and reperfusion. Neurogastroenterol. Motil..

[130] Giaroni C., Marchet S., Carpanese E., Prandoni V., Oldrini R., Bartolini B., Moro E., Vigetti D., Crema F., Lecchini S. (2013). Role of neuronal and inducible nitric oxide synthases in the guinea pig ileum myenteric plexus during in vitro ischemia and reperfusion. Neurogastroenterol. Motil..

[131] Lindeström L.-M., Ekblad E. (2004). Structural and neuronal changes in rat ileum after ischemia with reperfusion. Dig. Dis. Sci..

[132] Al’Qteishat A., Gaffney J., Krupinski J., Rubio F., West D., Kumar S., Kumar P., Mitsios N., Slevin M. (2006). Changes in hyaluronan production and metabolism following ischaemic stroke in man. Brain.

[133] Lindwall C., Olsson M., Osman A.M., Kuhn H.G., Curtis M.A. (2013). Selective expression of hyaluronan and receptor for hyaluronan mediated motility (Rhamm) in the adult mouse subventricular zone and rostral migratory stream and in ischemic cortex. Brain Res..

[134] Eldridge L., Moldobaeva A., Wagner E.M. (2011). Increased hyaluronan fragmentation during pulmonary ischemia. Am. J. Physiol.-Lung Cell. Mol. Physiol..

[135] Melin J., Hellberg O., Funa K., Hällgren R., Larsson E.G., Fellström B.C. (2006). Ischemia-Induced Renal Expression of Hyaluronan and CD44 in Diabetic Rats. Nephron Exp. Nephrol..

[136] Johnsson C., Tufveson G., Wahlberg J., Hällgren R. (1996). Experimentally-induced warm renal ischemia induces cortical accumulation of hyaluronan in the kidney. Kidney Int..

[137] Colombaro V., Jadot I., Declèves A.-E., Voisin V., Giordano L., Habsch I., Malaisse J., Flamion B., Caron N. (2015). Lack of hyaluronidases exacerbates renal post-ischemic injury, inflammation, and fibrosis. Kidney Int..

[138] Kultti A., Pasonen-Seppänen S., Jauhiainen M., Rilla K.J., Kärnä R., Pyöriä E., Tammi R.H., Tammi M.I. (2009). 4-Methylumbelliferone inhibits hyaluronan synthesis by depletion of cellular UDP-glucuronic acid and downregulation of hyaluronan synthase 2 and 3. Exp. Cell Res..

[139] Marsilio I., Caputi V., Latorre E., Cerantola S., Paquola A., Alcalde A.I., Mesonero J.E., O’Mahony S.M., Bertazzo A., Giaroni C. (2020). Oxidized phospholipids affect small intestine neuromuscular transmission and serotonergic pathways in juvenile mice. Neurogastroenterol. Motil..

[140] Cerantola S., Caputi V., Marsilio I., Ridolfi M., Faggin S., Bistoletti M., Giaroni C., Giron M.C. (2020). Involvement of Enteric Glia in Small Intestine Neuromuscular Dysfunction of Toll-Like Receptor 4-Deficient Mice. Cells.

[141] Wilkins T., Sequoia J., Jennings W., Dorn B. (2017). Probiotics for Gastrointestinal Conditions: A Summary of the Evidence. Am. Fam. Physicians.

[142] Zommiti M., Feuilloley M.G.J., Connil N. (2020). Update of Probiotics in Human World: A Nonstop Source of Benefactions till the End of Time. Microorganisms.

[143] Chapman T.M., Plosker G.L., Figgitt D.P. (2006). VSL#3 probiotic mixture: A review of its use in chronic inflammatory bowel diseases. Drugs.

[144] Banfi D., Moro E., Bosi A., Bistoletti M., Cerantola S., Crema F., Maggi F., Giron M.C., Giaroni C., Baj A. (2021). Impact of Microbial Metabolites on Microbiota–Gut–Brain Axis in Inflammatory Bowel Disease. Int. J. Mol. Sci..

[145] Liu Y., Tran D.Q., Rhoads J.M. (2018). Probiotics in Disease Prevention and Treatment. J. Clin. Pharmacol..

[146] Turco F., Sarnelli G., Cirillo C., Palumbo I., De Giorgi F., D’Alessandro A., Cammarota M., Giuliano M., Cuomo R. (2014). Enteroglial-derived S100B protein integrates bacteria-induced Toll-like receptor signalling in human enteric glial cells. Gut.

[147] Corridoni D., Pastorelli L., Mattioli B., Locovei S., Ishikawa D., Arseneau K.O., Chieppa M., Cominelli F., Pizarro T.T. (2012). Probiotic Bacteria Regulate Intestinal Epithelial Permeability in Experimental Ileitis by a TNF-Dependent Mechanism. PLoS ONE.

[148] Lee B., Lee J.H., Lee H.S., Bae E.A., Huh C.S., Ahn Y.T., Kim D.H. (2009). Glycosaminoglycan degradation-inhibitory lactic acid bacteria ameliorate 2,4,6-trinitrobenzenesulfonic acid-induced colitis in mice. J. Microbiol. Biotechnol..

[149] Lee H.S., Han S.Y., Bae E.A., Huh C.S., Ahn Y.T., Lee J.H., Kim D.H. (2008). Lactic acid bacteria inhibit proinflammatory cytokine expression and bacterial glycosaminoglycan degradation activity in dextran sulfate sodium-induced colitic mice. Int. Immunopharmacol..

[150] Panthavee W., Noda M., Danshiitsoodol N., Kumagai T., Sugiyama M. (2017). Characterization of Exopolysaccharides Produced by Thermophilic Lactic Acid Bacteria Isolated from Tropical Fruits of Thailand. Biol. Pharm. Bull..

[151] Di Cerbo A., Aponte M., Esposito R., Bondi M., Palmieri B. (2013). Comparison of the effects of hyaluronidase and hyaluronic acid on probiotics growth. BMC Microbiol..

[152] Zheng H., Gao M., Ren Y., Lou R., Xie H., Yu W., Liu X., Ma X. (2017). An improved pH-responsive carrier based on EDTA-Ca-alginate for oral delivery of *Lactobacillus rhamnosus* ATCC 53103. Carbohydr. Polym..

[153] Shaharuddin S., Muhamad I.I. (2015). Microencapsulation of alginate-immobilized bagasse with Lactobacillus rhamnosus NRRL 442: Enhancement of survivability and thermotolerance. Carbohydr. Polym..

[154] Xiao Y., Lu C., Liu Y., Kong L., Bai H., Mu H., Li Z., Geng H., Duan J. (2020). Encapsulation of Lactobacillus rhamnosus in Hyaluronic Acid-Based Hydrogel for Pathogen-Targeted Delivery to Ameliorate Enteritis. ACS Appl. Mater. Interfaces.

[155] Di Cerbo A., Palmieri B. (2013). Lactobacillus Paracasei subsp. Paracasei F19; a farmacogenomic and clinical update. Nutr. Hosp..

[156] Liu H., Cai Z., Wang F., Hong L., Deng L., Zhong J., Wang Z., Cui W. (2021). Colon-Targeted Adhesive Hydrogel Microsphere for Regulation of Gut Immunity and Flora. Adv. Sci..

